# Intra-ovarian inflammatory states and their associations with embryo quality in normal-BMI PCOS patients undergoing IVF treatment

**DOI:** 10.1186/s12958-023-01183-6

**Published:** 2024-01-11

**Authors:** Jie Shang, Siyu Wang, Aiyuan Wang, Fang Li, Jing Zhang, Jin Wang, Rui Lv, Haixia Chen, Xiaohuan Mu, Kai Zhang, Xiaohong Bai, Ye Tian

**Affiliations:** 1 Department of Gynecology and Obstetrics, Tianjin Medical University General Hospital, No.154, Anshan Road, Heping District, Tianjin, 300052 China; 2 Tianjin Key Laboratory of Female Reproductive Health and Eugenics, Tianjin Medical University General Hospital, Tianjin, China; 3 Key Laboratory of Immune Microenvironment and Disease (Ministry of Education), Tianjin Key Laboratory of Medical Epigenetics, Department of Biochemistry and Molecular Biology, School of Basic Medical Sciences, The Province and Ministry Co-sponsored Collaborative Innovation Center for Medical Epigenetics, Tianjin Medical University, Tianjin, China

**Keywords:** Polycystic ovary syndrome, Normal BMI, Intra-ovarian inflammatory states, Embryo quality, In vitro fertilization

## Abstract

**Background:**

Polycystic ovary syndrome (PCOS) is the main cause of anovulatory infertility in women of reproductive age, and low-grade chronic inflammation plays a key role in the occurrence and development of PCOS. However, obesity, as a likely confounding factor, can affect the inflammatory state of PCOS patients.

**Objective:**

The aim of this study was to comprehensively investigate intra-ovarian inflammatory states and their impact on embryo quality in PCOS patients with a normal BMI undergoing IVF treatment.

**Methods:**

DIA-mass spectrometry-based proteomics and bioinformatic analysis were combined to comprehensively profile the protein expression of granulosa cells (GCs) from 5 normal-BMI PCOS patients and 5 controls. Thirty-four cytokines were further systematically detected in follicular fluid (FF) from 32 age- and BMI-matched normal-BMI patients using Luminex liquid chip suspension technology. Next, the differentially expressed cytokines were evaluated by enzyme-linked immunosorbent assay (ELISA) in 24 newly recruited subjects, and the relationship between these cytokines and embryo quality in PCOS patients was analysed. Finally, these cytokine levels were compared and evaluated in PCOS patients with different androgen levels.

**Results:**

Proteomic analysis showed that the suppression of substance metabolism and steroid biosynthesis, more interestingly, resulted in an enhanced immune and inflammatory response in the GCs of normal-BMI PCOS patients and prompted the involvement of cytokines in this process. Luminex analysis further showed that FF macrophage inflammatory protein-1 beta (MIP-1β) and stromal cell-derived factor-1 alpha (SDF-1α) levels were significantly increased in normal-BMI PCOS patients compared to controls (*P* = 0.005; *P* = 0.035, respectively), and the ELISA results were consistent with these findings. Besides, FF MIP-1β showed an inverse correlation with the number of D3 good-quality embryos and the good-quality blastocyst rate in patients with PCOS (*P* = 0.006; *P* = 0.003, respectively), which remained significant after correction for multiple comparisons. Moreover, SDF-1α levels had no relationship with embryo development in PCOS patients. Additionally, SDF-1α levels were significantly lower in PCOS patients with high androgen levels than in controls (*P* = 0.031).

**Conclusions:**

Local ovarian inflammation was present in normal-BMI PCOS patients, affecting follicular development, and FF MIP-1β may be a potential biomarker associated with embryo quality in normal-BMI PCOS patients.

**Graphical abstract:**

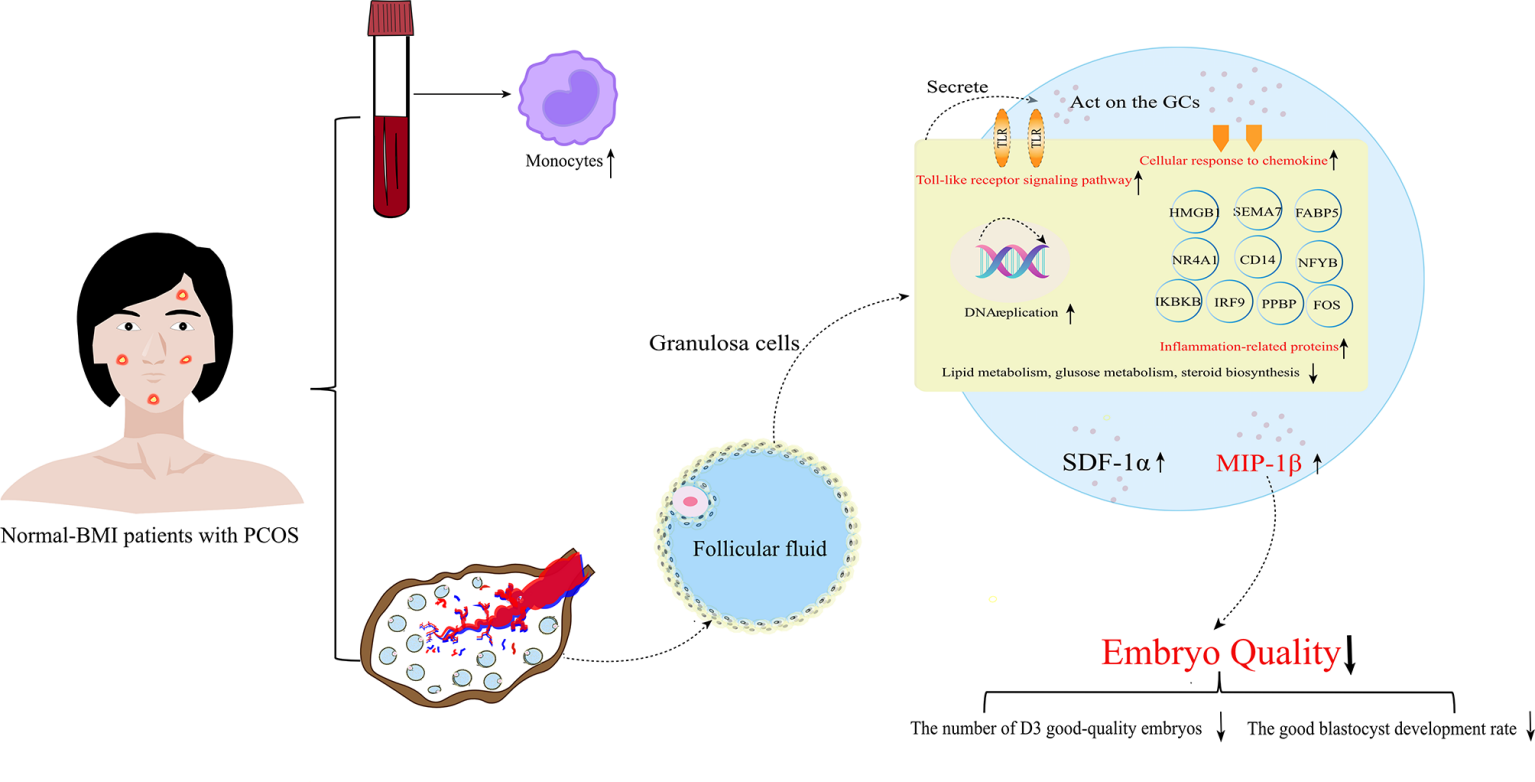

**Supplementary Information:**

The online version contains supplementary material available at 10.1186/s12958-023-01183-6.

## Introduction

Polycystic ovary syndrome (PCOS) is the most common gynaecological endocrine and metabolic disease in women of childbearing age. PCOS is characterized by ovulatory dysfunction, hyperandrogenism and polycystic ovarian morphology, with a global incidence of 5–20%, and is currently the main cause of anovulatory infertility [1]. Increasing evidence suggests that low-grade chronic inflammation plays a key role in the occurrence and development of PCOS, which is considered a heterogeneous disease. However, its specific role is not fully understood [2].

Numerous studies have showed a systemic low-grade chronic inflammatory state in women with PCOS and that the levels of cytokines (interleukin [IL]-6, IL-18, tumour necrosis factor-α [TNF-α]) and acute-phase proteins (high sensitivity C-reactive protein [hs-CRP], heat shock protein 70 [HSP70]) are increased in the peripheral blood [3]. The low-grade chronic inflammatory state in women with PCOS has been thought to be associated with the accumulation of visceral fat [4, 5]. More than 50% of PCOS patients are overweight or obese, while obesity induces adipotoxicity, leading to the release of large amounts of cytokines (serum C-reactive protein [CRP], TNF-α and IL-6) and adipokines into the systemic circulation and ovaries [6]. Therefore, obesity, as a likely confounding factor, can affect the inflammatory status of PCOS patients.

Low-grade chronic inflammation has been shown to affect ovarian function, ovulation, fertilization and embryo implantation in females with PCOS [7]. Abnormalities in oocyte and early embryo development and negative reproductive outcomes among obese subjects are also thought to be linked to inflammation [8, 9]. However, the potential for the development of oocytes and top-quality embryos tends to reduce in nonobese PCOS patients, and pregnancy outcomes also tend to be poorer [10, 11], although the cause is not clear.

The follicle is the basic functional unit of oocyte generation and development, and granulosa cells (GCs) and follicular fluid (FF) provide a very important microenvironment for follicular growth and oocyte maturation. GCs play a key role in the development of normal follicles, and abnormalities in GCs, such as increased levels of inflammation, can affect follicular development and are correlated with follicular atresia [12]. Besides, FF is closely connected to GCs. Recent work showed that cytokines are significant regulators of gamete production and steroidogenesis [13] that are present in FF, that they play diverse roles throughout folliculogenesis, and that they are intertwined with inflammation [14]. Elevated inflammatory cytokines in FF can affect GC function and interfere with normal oocyte development [15]. The above studies suggest that inflammatory changes in the local ovarian microenvironment are likely to be a key cause of abnormal follicular development. However, the inflammatory status of the local ovarian microenvironment in PCOS patients with a normal body mass index (BMI) remains to be explored.

Previous comprehensive studies of the inflammatory state in patients with PCOS have focused mainly on serological detection. However, fewer studies have focused on the inflammatory state of the local ovarian microenvironment, and others address only one or a few inflammatory proteins or cytokines of interest but with little assessment of the impact on embryo quality or developmental competence.

In this study, we hypothesized that a low-grade chronic inflammatory state exists in the ovaries of women with PCOS with a normal BMI and that it may affect embryo quality, as reflected comprehensively by the proteomic analysis of GCs and characteristics of the FF cytokine profile. Therefore, this study aimed to systematically investigate intra-ovarian inflammatory states and their impact on embryo quality in PCOS patients with a normal BMI undergoing in vitro fertilization (IVF) and to identify potential inflammatory markers that influence the developmental competence of embryos in PCOS patients with a normal BMI.

## Materials and methods

In this study, subjects who underwent IVF visiting the Reproductive Medical Center of Tianjin Medical University General Hospital from September 2022 to August 2023 were recruited. This study was approved and monitored by the Ethical Committee of Tianjin Medical University General Hospital (IRB2021-KY-196). All patients signed informed consent forms before starting the study.

### Patient selection

All included PCOS patients were diagnosed according to the 2003 Rotterdam diagnostic criteria and met two of the three following criteria: (1) oligo- or anovulation; (2) clinical and/or biochemical signs of hyperandrogenism; and (3) polycystic ovaries and exclusion of other etiologies (congenital adrenal hyperplasia, androgen-secreting tumors, Cushing’s syndrome) [16]. Each participant had a normal weight (18.5 kg/m^2^ ≤ BMI < 25 kg/m^2^) and was between 23 and 35 years old.

An age- and weight-matched control group of females was included in the study. These women had normal levels of free testosterone (T), no abnormalities on ovarian ultrasound examination, no hypertrichosis, and no other androgen-related skin manifestations.

The first exclusion criterion was the use of oral contraceptives or medications that may affect hormonal and metabolic levels within the last 6 months. To avoid the effects of some diseases on FF cytokines, we also excluded patients with metabolic disorders, known active infections or inflammatory diseases, autoimmune diseases, endometriosis, or diminished ovarian reserve.

### Clinical examination

The baseline hormonal and biochemical parameters of the women in the PCOS and control groups were measured. BMI (kg/m^2^) was calculated as weight (kg)/height squared (m^2^). Based on the World Health Organization (WHO)’s classification of BMI, 18.5 kg/m^2^ ≤ BMI < 25 kg/m^2^ was defined as normal weight [17]. Previous studies have shown that basal androgen levels ≥ 40 ng/dl in patients undergoing IVF are significantly detrimental to pregnancy outcomes [18]. Therefore, patients with PCOS were grouped according to basal serum androgen levels ≥ 40 ng/dl.

### IVF

All patients in our study received the appropriate controlled ovarian stimulation protocol with exogenous follicle-stimulating hormone (FSH) at individualized doses and according to their own situation. When the diameter of one or two follicles was ≥ 18 mm or those of three or more follicles were ≥ 17 mm, human chorionic gonadotropin (hCG) and/or the gonadotropin-releasing hormone (GnRH) agonist was administered to induce ovulation. After 36 h, oocytes were extracted by transvaginal ultrasound guidance.

On day 0, oocytes were fertilized by IVF or intracytoplasmic sperm injection (ICSI) and evaluated for the existence of both pronuclei (2PN) on day 1. Normal fertilization rates are 2PN%. On day 3, based on the Istanbul consensus, embryos were evaluated and graded [19]. On day 5 or 6, blastocysts were evaluated and graded according to the Gardner score [20].

### Follicular fluid acquisition and separation

After oocyte separation, the aspirated FF which wasn’t contaminated by visible blood from the first one to two mature follicles with a diameter of 17–20 mm was collected into the first 50 ml sterile centrifuge tube for subsequent detection of cytokines in the FF. And the remaining pooled FF sample from each patient was collected into the second and third 50 ml sterile centrifuge tubes for GC separation. At the same time, when collecting the FF, the FF containing visible blood was discarded to avoid blood contamination. The FF of the first 50 ml centrifuge tube centrifuged at room temperature at 2000 rpm for 10 min. The supernatant was separated and then stored in liquid nitrogen in a 2-ml cryogenic vial and used for the subsequent determination of the levels of various cytokines. FF collection for cryopreservation should be controlled within 30 min.

### Purification of human ovarian granulosa cells

GCs were collected from 5 PCOS patients and 5 controls for proteomic and bioinformatic analyses. The FF was collected in the above steps and centrifuged at 2000 rpm for 10 min at room temperature. The precipitate (including the resulting pellet of FF in the first tube) was resuspended with 2 ml of 100 IU/mL hyaluronidase (hyaluronidase from bovine testes type VIII H3757, Sigma–Aldrich, Merck KGaA, St Louis, USA) and transferred to a 15-mL centrifuge tube, followed by digestion at 37 °C for 40 min and mixing 3 times during this period. An equal volume of 1X Dulbecco’s phosphate buffered saline (DPBS) was added to mix well and left at room temperature for 10 min. A new 15-mL centrifuge tube was prepared, and a 3-mL lymphocyte separation solution (Ficoll) (Ficoll-Paque^TM^PLUS 17144003, Cytiva, Sweden, USA) was added. The 4-mL cell suspension was evenly spread on Ficoll followed by centrifugation at 1600 rpm for 10 min at room temperature. The cells in the middle layer of the separation solution were collected and resuspended in 1X DPBS and then centrifuged at 1800 rpm for 3 min at room temperature. The precipitate was resuspended in 1 mL of 1X DPBS, and 3 mL of red blood cell lysis buffer (Red BLOOD Cell Lysis Buffer, R1010, Solarbio® Science & Technology Co, Beijing, China) was added and mixed well, followed by 15 min on ice. The samples were then centrifuged at 450×g for 5 min at room temperature. The precipitate was resuspended in 1X DPBS, centrifuged at 1800 rpm for 3 min at room temperature and washed twice. The cells were then resuspended in 3 mL of 1X DPBS, divided equally into 2 cryogenic vials (2 mL), and centrifuged at 4500 rpm for 3 min at room temperature. The supernatant was discarded, and the cells were stored in liquid nitrogen.

### Protein extraction and digestion

First, we added RIPA buffer containing protease inhibitors into cryopreservation tubes containing GCs. After measuring the protein concentration using the BCA method, we prepared the protein solution with 100 µg proteins and added a 50% TCA solution to precipitate proteins. After washing twice with − 20 °C acetone, the protein pellets were dissolved in 100 mM NH_4_HCO_3_ (pH 8.0) for digestion. Protein solution was subjected to tryptic digestion at 37 °C for 16 h, and then DTT was added to 5 mM final concentration followed by incubation at 56 °C for 30 min. IAA was further added to alkylate proteins with 15 mM final concentration followed by incubation at room temperature in the dark for 30 min. The alkylation reaction was quenched by 30 mM cysteine (final concentration) at room temperature for another 30 min. Trypsin was again added at a trypsin-to-protein ratio of 1:100 (w/w) for digestion at 37 °C for 4 h. The resulting peptides were cleaned up with C18 ZipTips (Millipore), followed by LC-mass spectrometry (MS/MS) analysis.

### LC–MS/MS analysis

Peptides were dissolved in 0.1% FA and directly loaded onto a reversed-phase precolumn (Acclaim PepMap 100, Thermo Scientific). Peptide separation was performed using a reversed-phase analytical column (Acclaim PepMap RSLC, Thermo Scientific). The standard parameters commonly used in HPLC-MS/MS proteomics were performed. Specifically, the HPLC gradient elution for samples separation was performed by an increase from 5 to 22% solvent B (0.1% FA in 98% ACN) for 41 min, 22–38% for 23 min, climbing to 100% in 3 min and then holding at 100% for 8 min, all at a constant flow rate of 280 nl/min on an EASY-nLC 1000 UPLC system. The resulting peptides were injected into an NSI source followed by tandem MS/MS analysis in an Eclipse Orbitrap mass spectrometer (Thermo Fisher Scientific). Intact peptides were detected in the Orbitrap at a resolution of 60,000. For MS scans, the m/z scan range was 400 to 1200. A data-independent acquisition (DIA) procedure was performed with the setting parameters (Supplementary Table 1).

### Database search

The resulting MS/MS data were processed using Spectronaut. Tandem mass spectra were searched against UniProt human database (20,205 entries, downloaded in 2017.06.17) concatenated with a reverse decoy database. Trypsin/P was specified as a cleavage enzyme allowing up to 2 missing cleavages. The mass error was set to 10 ppm for precursor ions and 0.02 Da for fragment ions. Carbamidomethylation on cysteine was specified as a fixed modification, and oxidation on Met and acetylation on the protein N-terminus were specified as variable modifications. False discovery rate (FDR) thresholds for protein, peptide and modification sites were specified at 1%. The minimum peptide length was set at 7, and “match between runs” was enabled. All the other parameters in Spectranet were set to default values.

### Bioinformatic analysis

The bioinformatics analysis was performed using the R programming language. Data were stringently filtered to keep proteins with a maximum of 3 missing values in at least one condition. Missing values were imputed using the R package Impute (1.74.1) before statistical testing. Differential protein expression analysis was performed using a two-sided unpaired Student’s *t* test, and a *p* value < 0.05 was considered statistically significant. The volcano plot was created using the R package ggplot2 (v.3.4.3). Principal component analysis (PCA) was performed through the R packages FactoMineR (v.2.8) and factoextra (v.1.0.7). Gene set enrichment analysis (GSEA) was conducted by the clusterProfiler (v.4.8.3) package in R. The heatmap was generated using the pheatmap (1.0.12) package in R.

### Gene set enrichment analysis

To explore the biological signalling pathway, GSEA of the expression data was applied to assess enrichment of the KEGG as well as the GO gene sets. KEGG/GO pathways with significant enrichment results were further demonstrated on the basis of the normalized enrichment score (NES), FDR and *P* value. Gene sets with |NES|>1, *P* value < 0.05, and FDR q < 0.1 were finally considered significantly enriched [21].

### Quantification of cytokines in follicular fluid

Concentrations of 34 cytokines (IL-27, IL-1β, IL-2, IL-4, IL-8, IL-12p70, IL-13, IL-17 A, IL-31, interferon-γ [IFN-γ], granulocyte-macrophage colony-stimulating factor [GM-CSF], TNF-α, IFN-α, IL-9, TNF-β, IL-23, IL-15, IL-21, IL-22, IL-5, IL-10, growth-related oncogene-alpha [GRO-α)], IL-1α, IL-6, IL-7, IL-1RA, regulated on activation normal T-cell expressed and secreted [RANTES], IL-18, macrophage inflammatory protein-1alpha [MIP-1α], interferon-gamma-induced protein 10 [IP-10], eotaxin, monocyte chemotactic protein-1 [MCP-1], stromal cell-derived factor-1alpha [SDF-1α], and MIP-1β) in FF samples collected from 32 patients, including 16 PCOS and 16 non-PCOS patients, were quantified by Luminex xMAP technology using the Luminex® 100/200™ system (Thermo, #EPX340-12167-901).

### Enzyme-linked immunosorbent assay (ELISA)

According to the above inclusion and exclusion criteria, FF was collected from 24 recruited patients, including 12 PCOS and 12 non-PCOS patients, whose clinical characteristics are presented in Supplementary Table 2. SDF-1α and MIP-1β levels in the supernatants of isolated FF samples were evaluated by ELISA to verify the cytokine profile results. SDF-1α was quantified using a Human SDF-1α ELISA Kit (Quantikine® ELISA DSA00, R&D Systems Inc., MN, USA) following the manufacturer’s instructions. MIP-1β levels were quantified using the Human MIP-1β enzyme-linked immunosorbent assay kit (Quantikine® ELISA DMB00, R&D Systems Inc., MN, USA). All samples were removed from liquid nitrogen at the same time and thawed, detection was repeated two times, and the results are presented using the mean ± standard error of the mean (SEM). It was recommended to read the absorbance at a reference wavelength of 450 nm.

The minimum detectable dose (sensitivity) was 47.0 pg/mL for SDF-1α and 11.0 pg/mL for MIP-1β.

### Statistical analysis

Statistical analysis was performed using GraphPad Prism 9.4.0. The data were first tested for normality by the Kolmogorov–Smirnov test. An unpaired Student’s *t* test was used to compare two groups (means ± SEMs) when data conformed to a normal distribution. Otherwise, data were analysed with the Mann–Whitney *U* test. Categorical variables were compared by chi-square tests or Fisher’s exact probability method. Correlation analysis of SDF-1α and MIP-1β in FF with other clinical characteristics and laboratory parameters was performed with the Pearson test (normally distributed variables) or the Spearman test (non-normally distributed data). The hypothesis test was set to be two-sided. Probability values < 0.05 were considered statistically significant. The statistical methods and the significance criteria for proteomics analysis are listed in the corresponding method sections above.

## Results

### Clinical characteristics and laboratory parameters of 32 women with a normal BMI with or without PCOS

The demographic features, hormonal and biochemical levels and laboratory parameters of normal-BMI PCOS patients and controls were characterized, as shown in Table 1. There were no significant differences in age, infertility type (primary or secondary infertility), infertility years, basal FSH levels or BMI (*P* > 0.05). However, the circulating levels of luteinizing hormone (LH), LH/FSH ratio, free T, and anti-Müllerian hormone (AMH) were significantly higher in patients with PCOS (*P* < 0.05). In the serum, the white blood cell (WBC), neutrophil (NE) and lymphocyte counts were not obviously different between the groups (*P* > 0.05), while the monocyte count in the serum of women with PCOS was higher than that in the serum of the controls (*P* < 0.05).

Notably, the number of oocytes retrieved in women with PCOS was significantly higher than that retrieved in controls (*P* < 0.05). The rates of D3 good-quality embryos, high-quality blastocysts and blastocyst formation were not significantly different between the two groups (*P* > 0.05).

**Table 1:** Comparison of baseline clinical characteristics and laboratory parameters of normal-BMI women with and without PCOS

Group	PCOS group (*n* = 16)	Control group (*n* = 16)	P
Age (years)	30.31 ± 0.84	32.19 ± 0.53	NS
BMI (kg/m^2^)	22.38 ± 0.42	21.81 ± 0.45	NS
Primary infertility	10 (62.50%)	15 (93.75%)	NS
Secondary infertility	6 (37.50%)	1 (6.25%)	
Infertility years (years)	2.38 ± 0.36	2.63 ± 0.46	NS
FSH (IU/L)	5.63 ± 0.33	6.07 ± 0.39	NS
LH (IU/L)	7.11 ± 1.26	3.94 ± 0.52	0.027*
LH/FSH rate	1.14 ± 0.19	0.64 ± 0.06	0.018*
T (ng/dl)	39.99 ± 6.33	23.02 ± 3.28	0.023*
AMH (ng/ml)	8.16 ± 0.86	3.41 ± 0.34	<0.001*
WBC (10^9^/L)	6.56 ± 0.42	5.94 ± 0.43	NS
NE (10^9^/L)	3.87 ± 0.33	3.73 ± 0.45	NS
Lymphocytes (10^9^/L)	2.17 ± 0.18	1.79 ± 0.18	NS
Monocytes (10^9^/L)	0.41 ± 0.03	0.33 ± 0.02	0.030*
Ret. oocytes	29.25 ± 2.93	17.44 ± 1.85	0.002*
Rate of 2PN (%)	62.17 ± 3.58	58.10 ± 4.26	NS
D3 day high quality embryos	8.13 ± 1.33	5.06 ± 0.90	NS
Rate of D3 day quality embryo (%)	42.03 ± 4.86	54.83 ± 6.18	NS
Rate of high-quality blastocyst rate (%)	21.03 ± 6.36	28.90 ± 9.31	NS
Rate of blastocyst formation (%)	27.61 ± 5.30	34.76 ± 8.87	NS

BMI, body mass index; FSH, follicle-stimulating hormone; LH, luteinizing hormone; LH/FSH, luteinizing hormone/follicle-stimulating hormone; T, testosterone; AMH, anti-Müllerian hormone; WBC, white blood cell; NE, neutrophil; Ret. oocyte, number of oocytes retrieved; 2PN, 2 pronuclear; NS, not significant

Data are expressed as (means ± standard error of the mean (SEM); Count data are shown as a percentage (%); *Represents a statistically significant difference (*P* < 0.05)

### Proteomics analysis and inflammatory changes in granulosa cells from PCOS patients with a normal BMI

To obtain a more complete and comprehensive profile of the inflammatory state in granulosa cells from normal-BMI patients with PCOS, we applied mass spectrometry (MS) based on the DIA strategy to compare the proteomes of GCs from 5 normal-BMI PCOS patients and 5 controls matched for BMI and age; their clinical characteristics are presented in Supplementary Table 3. Compared to other traditional proteomics techniques, the DIA proteomics analysis used in this study has the advantages of high reproducibility, accuracy, quantitative stability, and broad protein coverage, and its combination with data analysis techniques could further improve assay performance [22, 23].

**Fig. 1:**
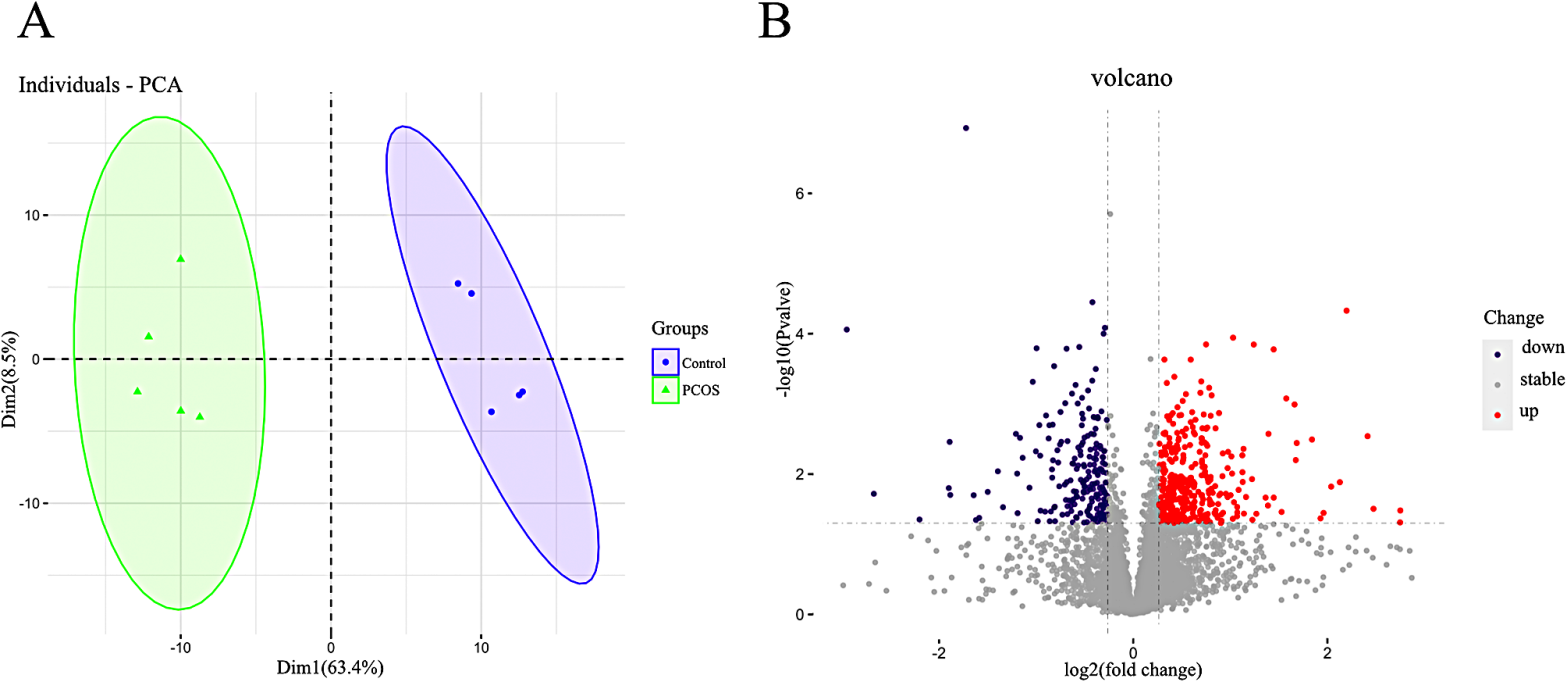
Global proteome analysis between the normal-BMI PCOS and control groups. (**A**) Principal component analysis (PCA) based on differential protein expression. The PCA plot shows the segregation of samples between the two groups based on proteomes. (**B**) Volcano plot of proteome quantification between the two groups. Red represents upregulated proteins, and purple represents downregulated proteins (p value < 0.05, |FC|≥1.2); FC, FoldChange

Through subsequent data processing, we identified a total of 5521 proteins. To obtain a global proteomic landscape of the GC from normal-BMI PCOS patients and control subjects, we performed the clustering analysis based on all identified proteins. PCA showed two significantly different clusters (Fig. 1A), suggesting a distinct difference in protein expression levels between the PCOS group and the control group. Subsequently, differentially expressed proteins (DEPs) were identified between the two groups with a 1.2-fold change as the threshold value and p value < 0.05 as the standard, and thus 356 proteins were found to be upregulated and 216 protein were found to be downregulated in the PCOS group compared to the control group (Fig. 1B). Notably, we found that a variety of inflammation-related proteins, such as CD14, FABP5, IKBKB, FOS, IRF9, NFYB, NR4A1 and SEMA7A, as well as proteins associated with cytokine and chemokine release, such as HMGB1 and PPBP (CXCL7), were upregulated in PCOS patients with a normal BMI (Supplementary Table 4).

**Fig. 2:**
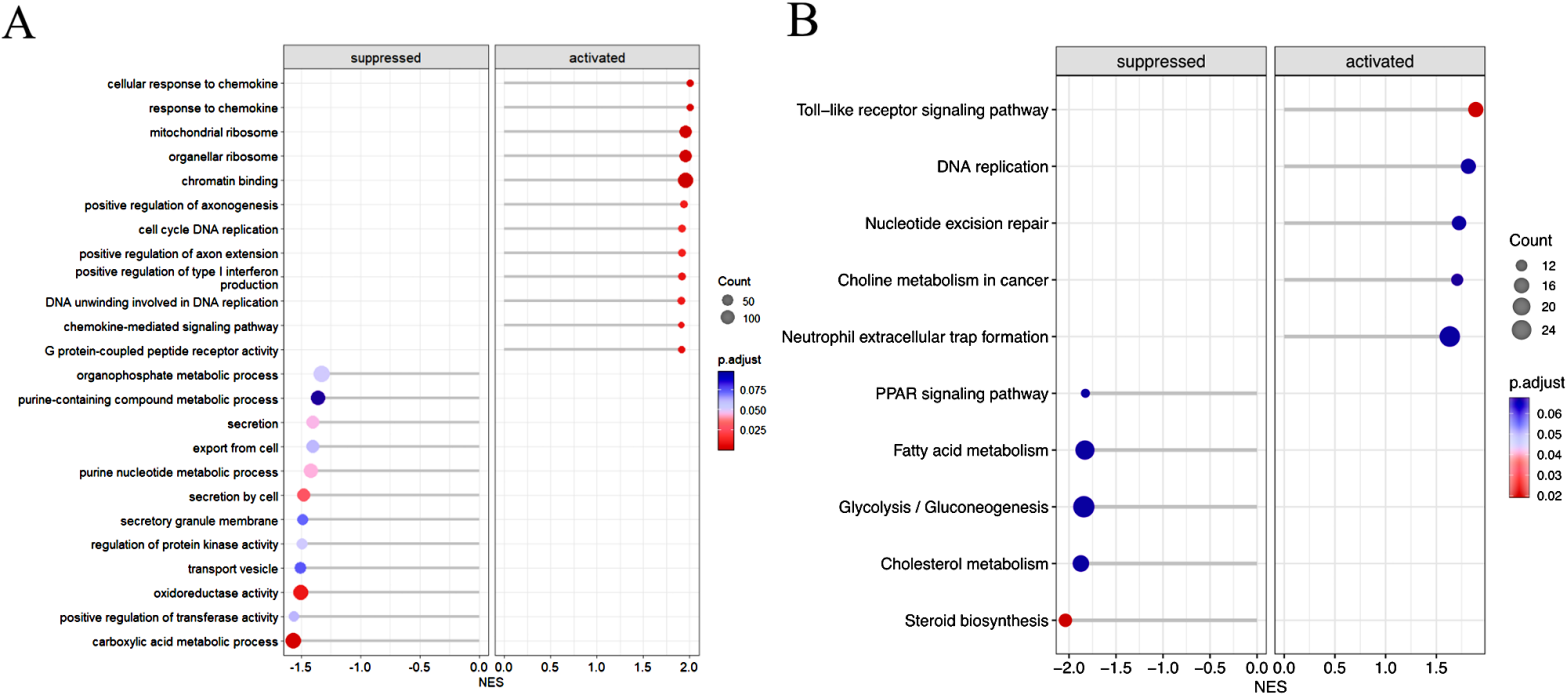
Gene Set Enrichment Analysis based on proteomes. (**A**) Functional enrichment of differential proteins by the Gene Ontology database (Left: Suppressed biological activities in the normal-BMI PCOS group; Right: Activated biological activities in the normal-BMI PCOS group). (**B**) Functional enrichment of differential proteins with KEGG pathway analysis (Left: Down KEGG pathway in the normal-BMI PCOS group; Right: Up KEGG pathway in the normal-BMI PCOS group)

Consistent with the above findings, KEGG/GO pathway-based GSEA also showed significant enrichment in immune and inflammatory signalling pathways such as “Toll-like receptor signalling pathway”, “cellular response to chemokine”, “chemokine-mediated signalling pathway”, and “response to chemokines” in the PCOS groups (*P* < 0.05). All of these signalling pathways were activated (Fig. 2A and B), suggesting an enhanced immune and inflammatory response in GCs from PCOS patients with a normal BMI compared to controls and a potential role for cytokines and chemokines in this process.

Besides, we found that signalling pathways were enriched in metabolism and cell cycle DNA replication and that the DNA replication signalling pathway was activated. Conversely, metabolic pathways, including lipid metabolism, glucose metabolism, nucleic acid metabolism and steroid biosynthesis, were obviously suppressed in the PCOS groups (Fig. 2A and B), suggesting that there are also abnormal changes in the metabolism and cell proliferation of GCs in PCOS patients with a normal BMI. The results will be confirmed in our future studies based on a larger cohort, as the sample size of the proteomic analysis in this study is limited.

### Follicular fluid 34 cytokine levels in normal-BMI PCOS participants and controls

**Fig. 3:**
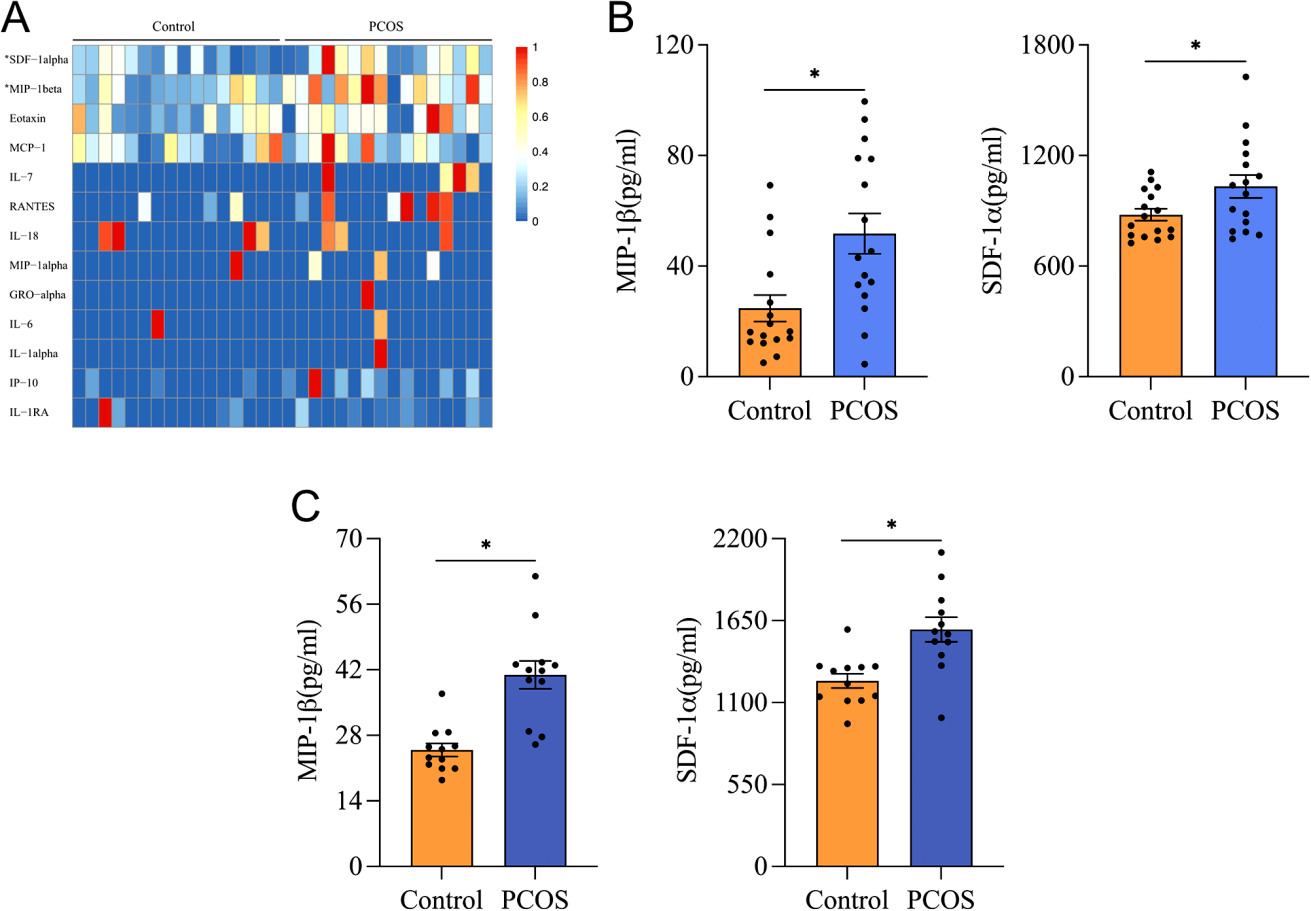
Analysis of the concentrations of 34 cytokines in follicular fluid by the Luminex liquid chip suspension technology assay in women with a normal body mass index with or without PCOS. (**A**) Hierarchical clustering of partial cytokine concentrations in the two groups of patients. Each row represents a kind of cytokine, and each column represents one sample. Samples from each group (*n* = 16) were arranged from left to right. The T test was used for SDF-1α. The remaining studies used the Mann–Whitney test. *P < 0.05 PCOS *versus* Control. (**B**) Concentrations of MIP-1β and SDF-1α in follicular fluid from women with a normal body mass index with or without PCOS measured by the Luminex. Each data point represents a specimen of one patient; *n* = 16 per group, all bars indicate the mean ± standard error of the mean, SDF-1α difference analysis followed by Student’s t test, MIP-1β difference analysis followed by the Mann–Whitney test, *P < 0.05. (**C**) Concentrations of MIP-1β and SDF-1α in follicular fluid from 24 women with a normal body mass index with or without PCOS measured by ELISA. Each data point represents a specimen of one patient; *n* = 12 per group, all bars indicate the mean ± standard error of the mean (SEM), difference analysis followed by Student’s t test, *P < 0.05

Given that the immune and inflammatory responses were enhanced in GCs from PCOS patients with a normal BMI according to proteomics analysis, cytokines and chemokines may play an important role in this process. In addition to the fact that FF, as a microenvironment for GCs, has a close connection with granulosa cells, we decided to systematically examine the cytokine signatures in the FF of PCOS patients with a normal BMI to obtain a more direct and comprehensive view of the FF inflammatory state. Therefore, we quantified and evaluated the levels of 34 cytokines in the FF of 32 (16 PCOS vs. 16 control) BMI-normal subjects (comprising 5 PCOS vs. 5 controls in proteomics analysis), whose clinical characteristics and laboratory parameters are presented in Table 1, using the Luminex liquid chip suspension technology assay, as shown in Fig. 3. Hierarchical clustering analysis of partial cytokine concentrations in the two groups showed that the normal-BMI PCOS group had significantly higher SDF-1α and MIP-1β levels in FF (mean: 1032.05 ± 61.69 vs. 878.57 ± 31.98 pg/ml; 51.78 ± 7.31 vs. 24.75 ± 4.78 pg/ml, respectively) (*P* < 0.05) (Fig. 3A and B). Consistently, the ELISA results showed that the concentrations of FF SDF-1α and MIP-1β were significantly increased in the PCOS group compared to the control group (Fig. 3C). Meanwhile, the levels of eotaxin and MCP-1 in the FF of PCOS patients tended to increase; however, the difference was not significant (*P* > 0.05). Additionally, we could not detect the other cytokines in the FF.

### SDF-1α and MIP-1β in FF and clinical characteristics of normal-BMI women with PCOS

**Fig. 4:**
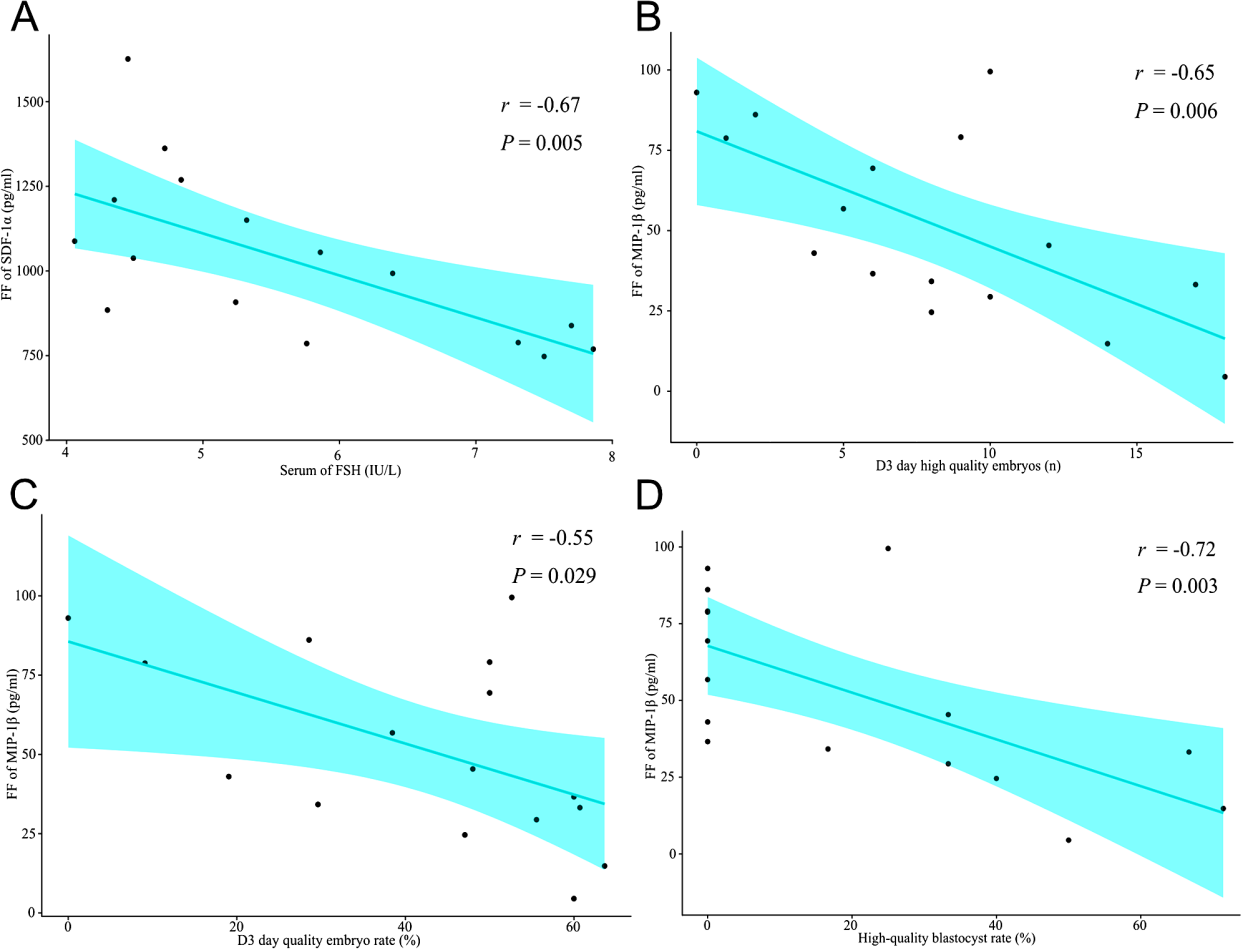
Correlation analysis between FF SDF-1α and MIP-1β levels and clinical and laboratory parameters in PCOS patients with a normal BMI. (**A**) Correlation of SDF-1α levels in follicular fluid with the serum FSH levels as determined by Pearson’s rank test (*P* = 0.005, *r* = −0.67, FDR = 0.070). (**B**) Correlation of MIP-1β levels in follicular fluid with D3 high-quality embryos as determined by Pearson’s rank test (*P* = 0.006, *r* = −0.65, FDR = 0.042). (**C**) Correlation of MIP-1β levels in follicular fluid with D3 good-quality embryo rate (%) as determined by Spearman’s rank test (*P* = 0.029, *r* = −0.55, FDR = 0.135). (**D**) Correlation of MIP-1β levels in follicular fluid with high-quality blastocyst rate (%) as determined by Spearman’s rank test (*P* = 0.003, *r* = −0.72, FDR = 0.042). Data represent a statistically significant difference (FDR<0.05)

Correlation analysis further showed that SDF-1α levels in the FF of PCOS patients with a normal BMI tended to be negatively correlated with FSH levels, but the correlation was not significant (*r* = −0.67, *P* = 0.005 (Fig. 4A); FDR = 0.070 (Supplementary Table 6)). Although the levels of SDF-1α and MIP-1β in the FF of women with PCOS were independent of parameters such as BMI, age, LH and T (Supplementary Table 5), compared with PCOS patients with lower T levels, FF SDF-1α was significantly lower in PCOS women with slightly higher T (mean: 1135 ± 76.66 pg/ml vs. 899.3 ± 80.32, *P* < 0.05) (Table 2). Besides, there was no significant correlation between follicular concentrations of SDF-1α and MIP-1β (r (95% CI) = 0.28 (-0.25 ~ 0.68), *P* = 0.293).

**Table 2:** FF SDF-1α, eotaxin, MCP-1 and MIP-1β levels based on T in normal-BMI PCOS women

Cytokines (pg/ml)	Lower T	Higher T	P
SDF-1α	1135 ± 76.66	899.30 ± 80.32	0.031*
Eotaxin	28.06 ± 4.91	14.90 ± 3.13	NS
MCP-1	73.07 ± 20.87	68.34 ± 24.06	NS
MIP-1β	57.51 ± 9.32	44.40 ± 11.83	NS

lower T: lower testosterone; higher T: higher testosterone; SDF-1α: stromal cell-derived factor-1 alpha; MCP-1: monocyte chemotactic protein-1; MIP-1β: macrophage inflammatory protein-1 beta; NS: not significant. Data are shown as the mean ± SEM. *Represents a statistically significant difference (*P* < 0.05)

### FF SDF-1α and MIP-1β and IVF in women with PCOS with a normal BMI

Finally, we observed a negative correlation among the level of FF MIP-1β in normal-BMI PCOS patients with D3 high-quality embryos (*r* = −0.65, *P* = 0.006 (Fig. 4B); FDR = 0.042 (Supplementary Table 6)) and the high-quality blastocyst rate (%) (*r* = −0.72 *P* = 0.003 (Fig. 4D); FDR = 0.042 (Supplementary Table 6). However, FF SDF-1α lacked relevance to Ret. Oocyte, D3 high-quality embryos, the D3 good-quality embryo rate (%) and the high-quality blastocyst rate (%) (*P* > 0.05) (Supplementary Table 5).

## Discussion

PCOS is closely associated with infertility. Prior studies have shown that systemic low-grade chronic inflammation is associated with PCOS and affects oocyte and early embryonic development. Meanwhile, recent research has also shown that low-grade chronic inflammation in the ovaries of PCOS patients is very important and may contribute to reproductive dysfunction. Simple obesity reduces fertility and is directly associated with poorer reproductive prognosis [24], possibly due to obesity-induced chronic inflammation of the ovaries [25]. Hence, in this study, the patients with a normal BMI were selected to remove the effect of the confounding factor of obesity on reproductive function in women with PCOS.

Oocyte quality and developmental competence are the main limitations of women’s reproductive capacity and GCs around the oocyte can reflect the characteristics of the oocyte [26]. Besides, inflammation in GCs can lead to granulosa cell dysfunction, which in turn affects oocyte quality and subsequent embryonic developmental competence [27]. Traditional research on inflammation in granulosa cells has used targeted approaches to one or a few proteins or signalling pathways, which do not allow an overall assessment of the inflammatory state of GCs. To obtain a more comprehensive profile of the inflammatory state of GCs, we combined the DIA-based proteomics, which is more reliable, accurate and broadest protein coverage compared to traditional proteomics technology, with a bioinformatic analysis to identify the proteomes of GCs from PCOS patients with a normal BMI undergoing IVF treatment and further found the suppression of substance metabolism (glucose metabolism, fatty acid metabolism, cholesterol metabolism) and steroid biosynthesis in GCs. More interestingly, we noticed that the expression of many inflammation-related proteins was upregulated in GCs and that several activated pathways were enriched in immunity and inflammation, such as the Toll-like receptor (TLR) signalling pathway, chemokine-mediated signalling pathway and the cellular response to chemokines, which are a subgroup of cytokines. Activated TLRs initiate downstream signalling to produce various types of cytokines, and as important signalling molecules, cytokines play important roles in the regulation of cell differentiation, proliferation, angiogenesis and the inflammatory response [28].

FF is the microenvironment that ensures the survival of GCs and is closely related to GCs. According to the proteomics results, we further systematically measured the levels of 34 cytokines in FF by cytokine chip technology to obtain a more complete understanding of the FF inflammatory state in PCOS patients with a normal BMI undergoing IVF treatment. To ensure the accuracy of the results at the same time, we investigated FF cytokine levels in two cohorts of samples from different subjects with the same inclusion and exclusion criteria by two methods. In our comparison of the levels of cytokines in the FF of the normal-BMI PCOS group and control group, the results showed significant differences in MIP-1β and SDF-1α between the two groups. Previous studies have showed that alterations in circulating cytokines can affect oocyte development. There was a certain correlation between metabolism in serum and FF in patients undergoing IVF treatment. Given that FF is less disrupted by other tissues and organs than serum, and in predicting embryo quality and developmental competence, changes in FF cytokines are more reliable than those in serum [29]. Therefore, we tried to identify a potential biomarker associated with embryo quality in PCOS patients with a normal BMI undergoing IVF treatment. Based on the above findings, we explored the correlation between MIP-1β or SDF-1α and embryo quality and found that MIP-1β was significantly associated with embryo quality. Our finding addresses the knowledge gap regarding the intra-ovarian inflammatory state and its association with embryo quality in PCOS patients with a normal BMI undergoing IVF treatment and reveals a potential biomarker associated with embryo quality in PCOS patients with a normal BMI.

In this study, we found that the levels of MIP-1β and SDF-1α in the normal-BMI PCOS group were significantly higher than those in the control group. MIP-1β, also known as C-C chemokine ligand 4 (CCL4), is a T-helper1 (Th1)-type CC chemokine that is produced mainly by activated monocytes and regulated by multiple cytokines [30]. In the present study, we also found elevated monocyte counts in the peripheral blood of normal-BMI patients with PCOS, which may be associated with elevated levels of MIP-1β in FF, and further studies are still needed in the future. MIP-1β has different effects on various types of immune and nonimmune cells under different pathophysiological conditions. There is evidence indicating that MIP-1β can promote the development of tumours by inducing protumorigenic macrophages, regulatory T-cell infiltration and other chemokines to suppress tumour immunity; conversely, MIP-1β can ramp up tumour immunity by recruiting macrophages with phagocytic ability and cytolytic lymphocytes [31]. SDF-1α (C-X-C motif ligand 12 (CXCL12)) was originally identified as a pre-B-cell growth factor (PBGF) and is considered the most potent chemokine because it can activate the migration and/or recruit a variety of leukocytes, including lymphocytes, monocytes, haematopoietic stem cells and progenitor cells [32]. It has previously been reported that FF MIP-1β levels are correlated with oocyte quality and pregnancy outcomes [33, 34]. In addition, in vitro experiments revealed that SDF-1α inhibits granulosa cell apoptosis in the KGN human ovarian granulosa cancer cell line in a dose-dependent manner [35] and plays an important role in embryogenesis [36].

Aberrant cytokines in FF can lead to abnormalities in folliculogenesis, oocyte quality and the embryo developmental capacity [37]. Several key parameters including normal fertilization rate (2PN%), the number of D3 good-quality embryos, the rate of D3 good-quality embryos, the good-quality blastocyst rate and the blastocyst formation rate, reflect the fertilization capacity of oocytes and embryonic developmental potential [19, 38]. And thus those indicators are often used to predict embryo quality [39]. Therefore, we chose the above metrics to study embryo quality and explored the correlation between the two cytokines and embryo quality. We further found that the number of D3 good-quality embryos and the rate of the good-quality blastocysts in patients with PCOS decreased remarkably with increasing levels of MIP-1β levels in FF. The data suggest that MIP-1β not only affects folliculogenesis but also mediates the mechanism that may impair oocyte maturation and embryonic development, which has a detrimental effect on embryo quality in patients with PCOS. Additionally, SDF-1α has been reported to maintain the number of follicles and improve ovarian reserve function by inhibiting follicle activation. In vitro addition of recombinant SDF-1α significantly increased follicle densities in the ovaries of neonatal mice, while the number of activated follicles in the ovaries was significantly reduced [40]. Besides, serum early-follicular-phase FSH has been referred to as a biomarker of ovarian reserve, and its elevation is strongly associated with premature ovarian insufficiency (POI) [41]. In this study, we found that SDF-1α levels tended to be negatively correlated with FSH, although the correlation was not significant. And SDF-1α levels had no relationship with embryo quality. This suggested to some extent that elevated SDF-1α levels may improve ovarian reserve but may also induce infertility in PCOS patients by inhibiting follicle activation rather than oocyte maturation and that the IVF process may mask the effects of SDF-1α on reproduction in PCOS patients, a speculation that needs to be confirmed by further studies.

Hyperandrogenism is considered to be the main cause of PCOS [42]. Androgen excess can result in FF microenvironment abnormalities, compromising oocyte developmental competence and leading to lower oocyte maturation rates [43, 44]. In this study, PCOS patients were divided into two groups based on androgen levels. We found that SDF-1α levels were significantly lower in PCOS patients with high androgen levels, while there was no significant difference in MIP-1β levels between the two groups, suggesting that SDF-1α is closely related to androgen levels and that it may play an important role in regulating reproductive function in PCOS patients.

A key step in IVF treatment is to assess the developmental competence of oocytes, embryos and blastocysts, and ultimately to identify the most viable embryos for transfer in order to achieve the highest pregnancy and live birth rates [26]. Current embryo assessment strategies rely heavily on morphological assessment of embryos and blastocysts and lack an objective and accurate biochemical indicator for oocyte and embryo selection [45]. In this study, FF MIP-1β was found to be associated with the number of D3 good-quality embryos, the rate of D3 good-quality embryos and the good-quality blastocyst rate, which may be a non-invasive indicator to assess embryo quality and viability in normal-BMI PCOS patients undergoing IVF treatment, and can be combined with the morphology score to optimize embryo selection to obtain the best embryos for transfer, which may lead to an increase in pregnancy and live birth rates and a decrease in miscarriage rates. And from a therapeutic perspective in the future, we believe that FF for MIP-1β detection can be performed at the same time as oocyte retrieval to predict the outcome of the embryo in advance, which may provide a window of time to take corrective or remedial measures to improve the quality of the embryo.

However, as this study only observed the correlation between the level of FF MIP-1β and embryo quality in normal-BMI PCOS patients undergoing IVF treatment, and it is not yet clear how it relates to pregnancy outcome, further studies are needed in the future. The sensitivity, specificity, positive predictive value and negative predictive value of MIP-1β for predicting embryo quality need further clarification. Moreover, this study is mainly relevant and exploratory, and it still needs to be further validated in animal experiments, in vitro experiments and large-scale, prospective and well-controlled randomised controlled trials.

## Conclusions

In this study, we revealed that local ovarian inflammation was present in PCOS patients with a normal BMI, affecting follicular development. By DIA-proteomics analysis, we observed the suppression of substance metabolism and steroid biosynthesis in GCs and, more interestingly, found the enhanced immune and inflammatory responses in GCs from PCOS patients with a normal BMI. We also noted the potential roles of cytokines and chemokines in this process. Thus, we comprehensively assessed 34 cytokines in FF and found that women with PCOS and a normal BMI have significantly elevated MIP-1β and SDF-1α levels in FF compared with control participants of similar age and BMI. Further analysis indicates MIP-1β as a potential biomarker associated with embryo quality in PCOS patients with a normal BMI. The study is a small number of cases in a single centre and needs to be extended in a future multicentre cohort study with a larger case.

### Electronic supplementary material

Below is the link to the electronic supplementary material.

Supplementary Material 1: The strategy to identify intra-ovarian inflammatory states and key factors in PCOS patients with a normal BMI undergoing IVF treatment

## Data Availability

Data available on request from the corresponding author.

## Abbreviations

FF: follicular fluid
GCs: granulosa cells
BMI: body mass index
ELISA: enzyme-linked immunosorbent assay
PCOS: polycystic ovary syndrome
MIP-1β: macrophage inflammatory protein-1 beta
SDF-1α: stromal cell-derived factor-1 alpha
FSH: follicle-stimulating hormone
IVF: in vitro fertilization
IL: interleukin
TNF-α: tumor necrosis factor-α
hs-CRP: high sensitivity C-reactive protein
HSP70: heat shock protein 70
CRP: serum C-reactive protein
WHO: the World Health Organization
hCG: human chorionic gonadotropin
GnRH: Gonadotrophin-releasing hormone
ICSI: intracytoplasmic sperm injection
2PN: both pronuclei
IFN-γ: interferon-γ
GM-CSF: granulocyte-macrophage colony-stimulating factor
GRO-α: growth-related oncogene-alpha
RANTES: regulated on activation normal T-cell expressed and secreted
MIP-1α: macrophage inflammatory protein-1alpha
IP-10: interferon-gamma-induced protein 10
MCP-1: monocyte chemotactic protein-1
SEM: standard error of the mean
WBC: white blood cell
NE: neutrophils
Ret. oocyte: number of oocytes retrieved
AMH: anti-Mllerian hormone
LH: luteinizing hormone
LH/FSH: luteinizing hormone/follicle stimulating hormone
T: testosterone
CCL4: C-C chemokine ligand 4
CXCL12: C-X-C motif ligand 12
PBGF: pre-B cell growth factor
POI: premature ovarian insufficiency
KGN: human ovarian granulosa tumor cell line
GSEA: gene set enrichment analysis
NES: normalized enrichment score
FDR: false discovery rate
MS: mass spectrometry
DIA: Data Independent Acquisition
PCA: Principal component analysis
DEPs: differentially expressed proteins
TLR: Toll-like receptor
FA: formic acid
ACN: acetonitrile
NS: not significant
FC: FoldChange

## References

[1] Azziz R, Carmina E, Chen Z, Dunaif A, Laven JSE, Legro RS (2016). Polycystic ovary syndrome. Nat Rev Dis Primers.

[2] Rudnicka E, Suchta K, Grymowicz M, Calik-Ksepka A, Smolarczyk K, Duszewska AM (2021). Chronic low Grade inflammation in Pathogenesis of PCOS. Int J Mol Sci.

[3] Zhai Y, Pang Y (2022). Systemic and ovarian inflammation in women with polycystic ovary syndrome. J Reprod Immunol.

[4] Escobar-Morreale HF (2018). Polycystic ovary syndrome: definition, aetiology, diagnosis and treatment. Nat Rev Endocrinol.

[5] Hu C, Pang B, Ma Z, Yi H (2020). Immunophenotypic profiles in polycystic ovary syndrome. Mediators Inflamm.

[6] Bahceci M, Gokalp D, Bahceci S, Tuzcu A, Atmaca S, Arikan S (2007). The correlation between adiposity and adiponectin, tumor necrosis factor alpha, interleukin-6 and high sensitivity C-reactive protein levels. Is adipocyte size associated with inflammation in adults?. J Endocrinol Invest.

[7] Yang Z, Tang Z, Cao X, Xie Q, Hu C, Zhong Z (2020). Controlling chronic low-grade inflammation to improve follicle development and survival. Am J Reprod Immunol.

[8] Cardozo E, Pavone ME, Hirshfeld-Cytron JE (2011). Metabolic syndrome and oocyte quality. Trends Endocrinol Metab.

[9] Kumbak B, Oral E, Bukulmez O (2012). Female obesity and assisted reproductive technologies. Semin Reprod Med.

[10] Sayutti N, Abu MA, Ahmad MF (2022). PCOS and role of Cumulus Gene expression in assessing oocytes Quality. Front Endocrinol (Lausanne).

[11] Rehman R, Mehmood M, Ali R, Shaharyar S, Alam F (2018). Influence of body mass index and polycystic ovarian syndrome on ICSI/IVF treatment outcomes: a study conducted in Pakistani women. Int J Reprod Biomed.

[12] Boots CE, Jungheim ES (2015). Inflammation and human ovarian Follicular dynamics. Semin Reprod Med.

[13] Hammadeh ME, Fischer-Hammadeh C, Amer AS, Rosenbaum P, Schmidt W (2005). Relationship between cytokine concentration in serum and preovulatory follicular fluid and in vitro fertilization/intracytoplasmic sperm injection outcome. Chem Immunol Allergy.

[14] Aydogan Mathyk B, Quaas AM (2021). Obesity and IVF: weighing in on the evidence. J Assist Reprod Genet.

[15] Liu Y, Liu H, Li Z, Fan H, Yan X, Liu X (2021). The release of Peripheral Immune Inflammatory cytokines promote an inflammatory Cascade in PCOS patients via altering the Follicular Microenvironment. Front Immunol.

[16] Rotterdam ESHRE, ASRM-Sponsored PCOS consensus workshop group (2004). Revised 2003 consensus on diagnostic criteria and long-term health risks related to polycystic ovary syndrome (PCOS). Hum Reprod.

[17] Obesity (2000). Preventing and managing the global epidemic. Report of a WHO consultation. World Health Organ Tech Rep Ser.

[18] Pan D, Shi J, Zhou H, Li N, Qu P (2018). Predictive value of basal androgen levels on ongoing pregnancy rates during in vitro fertilization cycles. Gynecol Endocrinol.

[19] Alpha Scientists in Reproductive Medicine and ESHRE Special Interest Group of Embryology. The Istanbul consensus workshop on embryo assessment: proceedings of an expert meeting. Hum Reprod. 2011;26:1270–83. 10.1093/humrep/der037 21502182

[20] Gardner DK, Lane M, Stevens J, Schlenker T, Schoolcraft WB (2000). Blastocyst score affects implantation and pregnancy outcome: towards a single blastocyst transfer. Fertil Steril.

[21] Subramanian A, Tamayo P, Mootha VK, Mukherjee S, Ebert BL, Gillette MA (2005). Gene set enrichment analysis: a knowledge-based approach for interpreting genome-wide expression profiles. Proc Natl Acad Sci U S A.

[22] Li J, Smith LS, Zhu H-J (2021). Data-independent acquisition (DIA): an emerging proteomics technology for analysis of drug-metabolizing enzymes and transporters. Drug Discov Today Technol.

[23] Zeng H-L, Hu L, Chen X, Han Q-Q, Li H, Cheng L (2023). DIA-MS based Proteomics combined with RNA-Seq Data to unveil the mitochondrial dysfunction in human glioblastoma. Molecules.

[24] Talmor A, Dunphy B (2015). Female obesity and infertility. Best Pract Res Clin Obstet Gynaecol.

[25] Robker RL, Akison LK, Bennett BD, Thrupp PN, Chura LR, Russell DL (2009). Obese women exhibit differences in ovarian metabolites, hormones, and gene expression compared with moderate-weight women. J Clin Endocrinol Metab.

[26] Uyar A, Torrealday S, Seli E (2013). Cumulus and granulosa cell markers of oocyte and embryo quality. Fertil Steril.

[27] Xie Q, Hong W, Li Y, Ling S, Zhou Z, Dai Y (2023). Chitosan oligosaccharide improves ovarian granulosa cells inflammation and oxidative stress in patients with polycystic ovary syndrome. Front Immunol.

[28] Patra MC, Shah M, Choi S (2020). Toll-like receptor-induced cytokines as immunotherapeutic targets in cancers and autoimmune diseases. Semin Cancer Biol.

[29] Hood RB, Liang D, Tan Y, Ford J, Souter I, Jones DP (2022). Characterizing the follicular fluid metabolome: quantifying the correlation across follicles and differences with the serum metabolome. Fertil Steril.

[30] Menten P, Wuyts A, Van Damme J (2002). Macrophage inflammatory protein-1. Cytokine Growth Factor Rev.

[31] Mukaida N, Sasaki S-I, Baba T (2020). CCL4 signaling in the Tumor Microenvironment. Adv Exp Med Biol.

[32] Janssens R, Struyf S, Proost P (2018). The unique structural and functional features of CXCL12. Cell Mol Immunol.

[33] Ostanin AA, Aizikovich BI, Aizikovich IV, Kozhin AY, Chernykh ER (2007). Role of cytokines in the regulation of reproductive function. Bull Exp Biol Med.

[34] Sarapik A, Velthut A, Haller-Kikkatalo K, Faure GC, Béné M-C, de Carvalho Bittencourt M (2012). Follicular proinflammatory cytokines and chemokines as markers of IVF success. Clin Dev Immunol.

[35] Jin L, Ren L, Lu J, Wen X, Zhuang S, Geng T (2021). CXCL12 and its receptors regulate granulosa cell apoptosis in PCOS rats and human KGN tumor cells. Reproduction.

[36] McGrath KE, Koniski AD, Maltby KM, McGann JK, Palis J (1999). Embryonic expression and function of the chemokine SDF-1 and its receptor, CXCR4. Dev Biol.

[37] Gaafar TM, Hanna MOF, Hammady MR, Amr HM, Osman OM, Nasef A (2014). Evaluation of cytokines in follicular fluid and their effect on fertilization and pregnancy outcome. Immunol Invest.

[38] The Vienna consensus. Report of an expert meeting on the development of ART laboratory performance indicators. Reproductive biomedicine online [Internet]. 2017 [cited 2023 Aug 28];35. Available from: https://pubmed.ncbi.nlm.nih.gov/28784335/. 10.1016/j.rbmo.2017.06.015 28784335

[39] Sunkara SK (2023). Number of oocytes and IVF outcomes: real-world evidence. Best Pract Res Clin Obstet Gynaecol.

[40] Holt JE, Jackson A, Roman SD, Aitken RJ, Koopman P, McLaughlin EA (2006). CXCR4/SDF1 interaction inhibits the primordial to primary follicle transition in the neonatal mouse ovary. Dev Biol.

[41] Steiner AZ, Pritchard D, Stanczyk FZ, Kesner JS, Meadows JW, Herring AH (2017). Association between Biomarkers of Ovarian Reserve and Infertility among Older Women of Reproductive Age. JAMA.

[42] Rodriguez Paris V, Bertoldo MJ (2019). The mechanism of androgen actions in PCOS Etiology. Med Sci (Basel).

[43] Patel SS, Carr BR (2008). Oocyte quality in adult polycystic ovary syndrome. Semin Reprod Med.

[44] Abbott DH, Padmanabhan V, Dumesic DA (2006). Contributions of androgen and estrogen to fetal programming of ovarian dysfunction. Reprod Biol Endocrinol.

[45] McKenzie LJ, Pangas SA, Carson SA, Kovanci E, Cisneros P, Buster JE (2004). Human cumulus granulosa cell gene expression: a predictor of fertilization and embryo selection in women undergoing IVF. Hum Reprod.

