# Supplementary material for: Intra-ovarian inflammatory states and their associations with embryo quality in normal-BMI PCOS patients undergoing IVF treatment
Source: Reprod Biol Endocrinol. 2024 Jan 11;22:11. doi: 10.1186/s12958-023-01183-6 (PMC10782707; doi:10.1186/s12958-023-01183-6)
Supplement: Supplementary file 1 — Supplementary Material 1: The strategy to identify intra-ovarian inflammatory states and key factors in PCOS patients with a normal BMI undergoing IVF treatment [file 12958_2023_1183_MOESM1_ESM.docx]

**
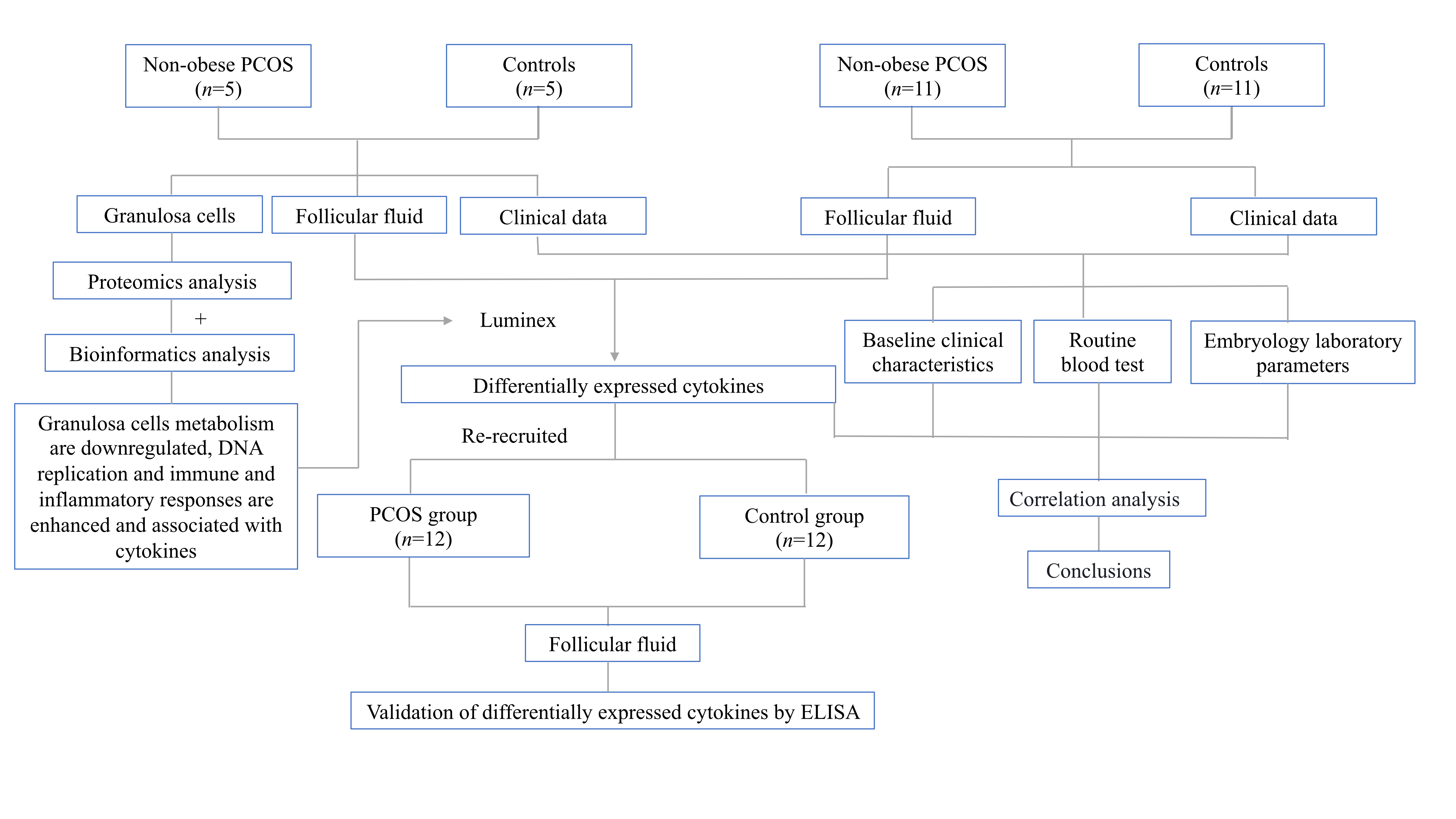
**

**Supplementary figure 1. The flowchart of the strategy to this work.**

**
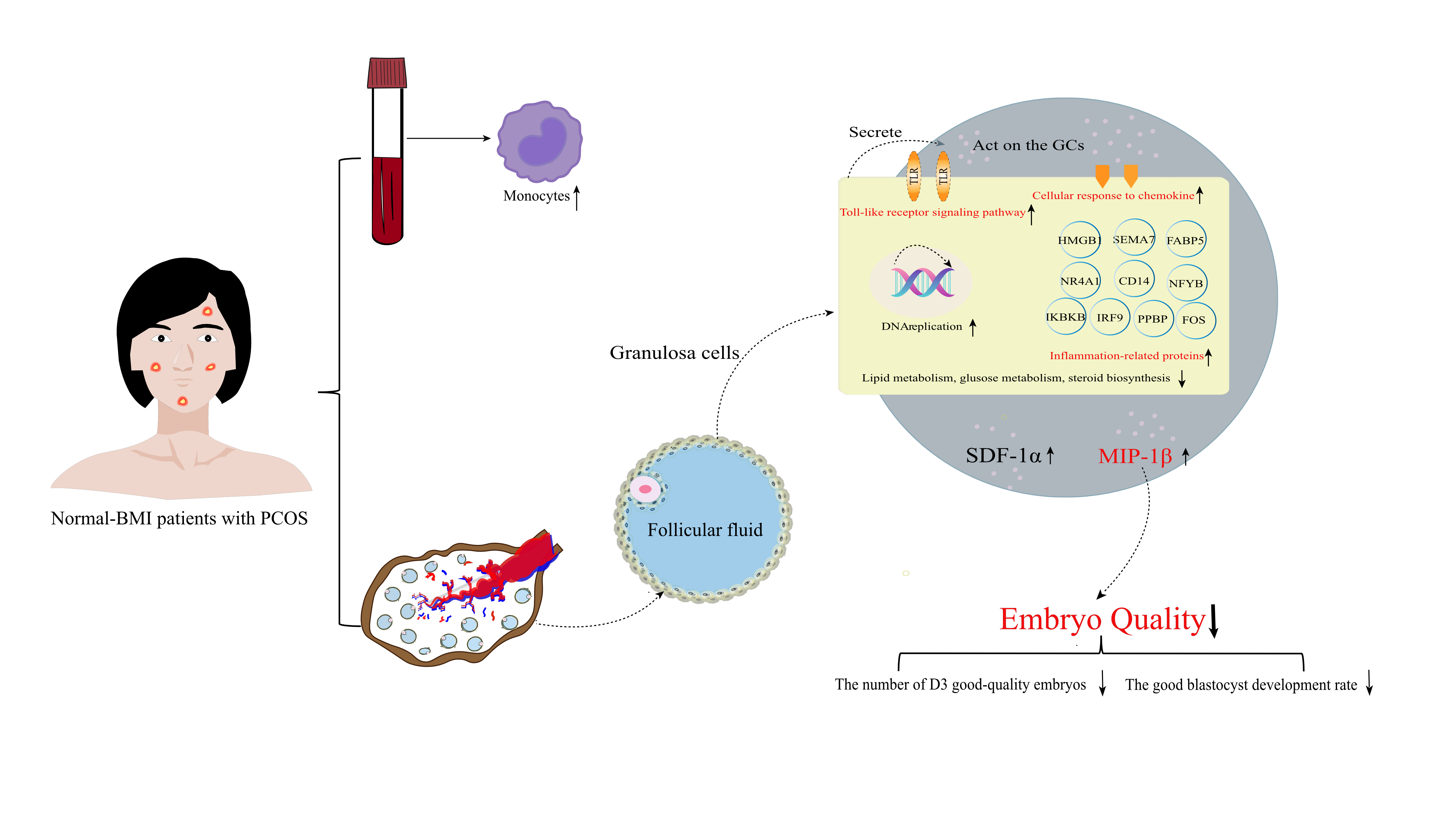
**

**Supplementary figure 2. The diagram of the strategy to this work.**

**Supplementary Table 1****. The parameters for tMS^2^ OT HCD**

| Targeted MS^n^ Scan Properties | Settings |
| --- | --- |
| MS^n^ Level (n) | 2 |
| Isolation Mode | Quadrupole |
| Activation Type | HCD |
| Collision Energy Mode | Fixed |
| HCD Collision Energy Type | Normalized |
| HCD Collision Energy (%) | 30 |
| Detector Type | Orbitrap |
| Orbitrap Resolution | 30000 |
| Mass Range | Normal |
| Scan Range Mode | Auto |
| RF Lens (%) | 40 |
| AGC Target | Custom |
| Nomalized AGC Target (%) | 1000 |
| Maximum Injection Time Mode | Custom |
| Maximum Injection Time (ms) | 100 |
| Microscans | 1 |
| Data Type | Centroid |
| Polarity  Loop Control  N (Number of Spectral)  Dynamic Retention Time  Time Mode | Positive  N  19  Off  Unscheduled |

Standard parameters in using HPLC-MS/MS Proteomics for DIA. Mass detector is Orbitrap while the mass spectrum acquisition mode is positive ion mode. The peptides were detached with HCD (High Energy Collision Dissociation).

**Supplementary Table 2.** **Clinical characteristics of women with** **a** **normal BMI with and without PCOS for ELISA.**

| Group | PCOS group  (*n*=12) | Control group  (*n*=12) | *P* |
| --- | --- | --- | --- |
| Age (years) | 30.33±0.86 | 31.42±0.68 | 0.242 |
| BMI (kg/m^2^) | 21.91±0.58 | 21.25±0.37 | 0.351 |
| Infertility years (years) | 3.58±0.67 | 2.33±0.41 | 0.133 |
| FSH (IU/L) | 5.66±0.31 | 6.11±0.52 | 0.745 |
| LH (IU/L) | 7.20±1.29 | 4.54±0.42 | 0.066 |
| LH/FSH rate | 1.32±0.25 | 0.79±0.08 | 0.028* |
| T (ng/dl) | 37.08±6.94 | 17.61±5.15 | 0.035* |
| AMH (ng/ml) | 8.09±1.23 | 4.33±0.56 | 0.011* |

ELISA: enzyme-linked immunosorbent assay; BMI: body mass index; FSH: follicle stimulating hormone; LH: luteinizing hormone; LH/FSH: luteinizing hormone/follicle stimulating hormone; T: testosterone; AMH: anti-M$\ddot{u}$llerian hormone; Data are expressed as (means ± standard error of the mean (SEM); ^*^Data represent a statistically significant difference(P<0.05).

**Supplementary Table 3.** **Clinical characteristics of women with** **a** **normal BMI with and without PCOS.**

| Group | PCOS group  (*n*=5) | Control group  (*n*=5) | *P* |
| --- | --- | --- | --- |
| Age (years) | 30.40±1.81 | 32.20±1.32 | 0.444 |
| BMI (kg/m^2^) | 23.00±0.85 | 22.32±0.73 | 0.561 |
| Infertility years (years) | 3.00±0.71 | 2.40±0.51 | 0.511 |
| FSH (IU/L) | 5.26±0.40 | 6.19±0.69 | 0.151 |
| LH (IU/L) | 5.15±1.21 | 4.16±1.10 | 0.560 |
| LH/FSH rate | 1.01±0.28 | 0.67±0.15 | 0.313 |
| T (ng/dl) | 36.78±19.02 | 27.85±4.43 | 0.841 |
| AMH (ng/ml) | 8.42±2.31 | 2.96±0.70 | 0.054 |

BMI: body mass index; FSH: follicle stimulating hormone; LH: luteinizing hormone; LH/FSH: luteinizing hormone/follicle stimulating hormone; T: testosterone; AMH: anti-M$\ddot{u}$llerian hormone; Data are expressed as (means ± standard error of the mean (SEM); ^*^Data represent a statistically significant difference(P<0.05).

**Supplementary Table 4. Inflammation-related upregulated differentially expressed proteins in PCOS patients with a normal BMI.**

| Symol | FoldChange | Log2 FC | P-Value |
| --- | --- | --- | --- |
| CD14 | 6.73 | 2.75 | 0.033^*^ |
| FABP5 | 1.21 | 0.28 | 0.046^*^ |
| FOS | 1.54 | 0.62 | 0.032^*^ |
| HMGB1 | 1.25 | 0.32 | 0.004^*^ |
| IKBKB | 1.49 | 0.57 | 0.031^*^ |
| IRF9 | 1.64 | 0.71 | 0.026^*^ |
| NFYB | 1.64 | 0.71 | 0.002^*^ |
| NR4A1 | 1.54 | 0.63 | 0.021^*^ |
| PPBP | 4.37 | 2.13 | 0.013^*^ |
| SEMA7A | 2.01 | 1.07 | 0.035^*^ |

NOTE. From external sets that support the listed candidates. Log FC and P value refer to results of the DIA analysis. ^*^Data represent a statistically significant difference(P<0.05) and |FC|≥1.2.

**Supplementary Table** **5. Correlation analysis between FF SDF-1α, MIP-1β levels and clinical and laboratory parameters in normal-BMI PCOS.**

| **Independent variables** | **SDF-1α (*n*=16)** |  | **MIP-1β (*n*=16)** |  |
| --- | --- | --- | --- | --- |
|  | **r (95%CI)/*P*** |  | **r (95%CI)/*P*** |  |
| age | 0.03(-0.47~0.52) 0.914 | | 0.22(-0.31~0.64) 0.415 | |
| BMI | -0.02(-0.51~0.48) 0.944 | | -0.09(-0.56~0.42) 0.729 | |
| FSH | -0.67(-0.87~-0.26)0.005* | | -0.17(-0.61~0.36) 0.529 | |
| LH | -0.43(-0.76~0.08) 0.097 | | 0.08(-0.43~0.56) 0.755 | |
| LH/FSH | -0.11(-0.58~0.41) 0.680 | | 0.09(-0.42~0.56) 0.733 | |
| T | -0.38(-0.74~0.14) 0.146 | | -0.24(-0.66~0.29) 0.372 | |
| AMH | -0.01 (-0.51~0.49) 0.959 | | -0.36(-0.73~0.16) 0.170 | |
| Monocytes | 0.27(-0.26~0.67) 0.316 | | -0.12(-0.58~0.40) 0.668 | |
| Ret. oocyte | -0.25(-0.67~0.28) 0.342 | | -0.50(-0.80~-0.002) 0.050 | |
| 2PN% | -0.05(-0.53~0.46) 0.852 | | -0.42(-0.76~0.10) 0.110 | |
| D3 day high quality embryos | -0.18(-0.62~0.34) 0.498 | | -0.65(-0.87~-0.23) 0.006* | |
| D3 day quality embryo rate (%) | -0.21(-0.65~0.33) 0.431 | | -0.55(-0.83~-0.06) 0.029* | |
| High-quality blastocyst rate (%) | -0.16(-0.62~0.38) 0.553 | | -0.72(-0.90~-0.33) 0.003* | |
| Blastocyst formation rate (%) | 0.46(-0.05~0.78) 0.074 | | -0.44(-0.77~0.08) 0.091 | |

BMI: body mass index; FSH: follicle stimulating hormone; LH: luteinizing hormone; LH/FSH: luteinizing hormone/follicle stimulating hormone; T: testosterone; AMH: anti-M$\ddot{u}$llerian hormone; Ret. oocyte: number of oocytes retrieved; 2PN: 2 pronuclear; *r*: Correlation coefficient; a positive value of *r* indicates a positive correlation, and a negative value of *r* indicates a negative correlation. 95%CI: 95% Confidence interval; ^*^Data represent a statistically significant difference(P<0.05).

**Supplementary Table 6. Results after correction for multiple comparisons for correlation analysis between FF SDF-1α, MIP-1β levels and clinical and laboratory parameters in normal-BMI PCOS.**

| **Independent variables** | **SDF-1α (*n*=16)** |  | **MIP-1β (*n*=16)** |  |
| --- | --- | --- | --- | --- |
|  | ***P*/FDR** |  | ***P*/FDR** |  |
| age | 0.914/ 0.959 | | 0.415/ 0.646 | |
| BMI | 0.944/ 0.959 | | 0.729/ 0.755 | |
| FSH | 0.005/ 0.070 | | 0.529/ 0.741 | |
| LH | 0.097/ 0.453 | | 0.755/ 0.755 | |
| LH/FSH | 0.680/ 0.952 | | 0.733/ 0.755 | |
| T | 0.146/ 0.511 | | 0.372/ 0.646 | |
| AMH | 0.959/ 0.959 | | 0.170/ 0.340 | |
| Monocytes | 0.316/ 0.798 | | 0.668/ 0.755 | |
| Ret. oocyte | 0.342/ 0.798 | | 0.050/ 0.175 | |
| 2PN% | 0.852/ 0.959 | | 0.110/ 0.257 | |
| D3 day high quality embryos | 0.498/ 0.860 | | 0.006/ 0.042* | |
| D3 day quality embryo rate (%) | 0.431/ 0.860 | | 0.029/ 0.135 | |
| High-quality blastocyst rate (%) | 0.553/ 0.860 | | 0.003/ 0.042* | |
| Blastocyst formation rate (%) | 0.074/ 0.453 | | 0.091/ 0.255 | |

BMI: body mass index; FSH: follicle stimulating hormone; LH: luteinizing hormone; LH/FSH: luteinizing hormone/follicle stimulating hormone; T: testosterone; AMH: anti-M$\ddot{u}$llerian hormone; Ret. Oocyte: number of oocytes retrieved; 2PN: 2 pronuclear; *P*: unadjusted *P* value; FDR: false discovery rate; ^*^Data represent a statistically significant difference (FDR<0.05).
